# Antibacterial activity and characteristics of silver nanoparticles biosynthesized from *Carduus crispus*

**DOI:** 10.1038/s41598-021-00520-2

**Published:** 2021-10-26

**Authors:** Enerelt Urnukhsaikhan, Bum-Erdene Bold, Aminaa Gunbileg, Nominchimeg Sukhbaatar, Tsogbadrakh Mishig-Ochir

**Affiliations:** Laboratory of Molecular and Cellular Biophysics, Department of Biology, National University of Mongolia, Ulaanbaatar, Mongolia

**Keywords:** Antimicrobials, Nanobiotechnology, Nanoscale materials

## Abstract

In recent years’ synthesis of metal nanoparticle using plants has been extensively studied and recognized as a non-toxic and efficient method applicable in biomedical field. The aim of this study is to investigate the role of different parts of medical plant *Carduus crispus* on synthesizing silver nanoparticles and characterize the produced nanoparticle. Our study showed that silver nanoparticles (AgNP) synthesized via whole plant extract exhibited a blue shift in absorption spectra with increased optical density, which correlates to a high yield and small size. Also, the results of zeta potential, X-ray diffraction, photon cross-correlation spectroscopy analysis showed the surface charge of − 54.29 ± 4.96 mV (AgNP-S), − 42.64 ± 3.762 mV (AgNP-F), − 46.02 ± 4.17 mV (AgNP-W), the crystallite size of 36 nm (AgNP-S), 13 nm (AgNP-F), 14 nm (AgNP-W) with face-centered cubic structure and average grain sizes of 145.1 nm, 22.5 nm and 99.6 nm. Another important characteristic, such as elemental composition and constituent capping agent has been determined by energy-dispersive X-ray spectroscopy and Fourier transform infrared. The silver nanoparticles were composed of ~ 80% Ag, ~ 15% K, and ~ 7.5% Ca (or ~ 2.8% P) elements. Moreover, the results of the FTIR measurement suggested that the distinct functional groups present in both AgNP-S and AgNP-F were found in AgNP-W. The atomic force microscopy analysis revealed that AgNP-S, AgNP-F and AgNP-W had sizes of 131 nm, 33 nm and 70 nm respectively. In addition, the biosynthesized silver nanoparticles were evaluated for their cytotoxicity and antibacterial activity. At 17 µg/ml concentration, AgNP-S, AgNP-F and AgNP-W showed very low toxicity on HepG2 cell line but also high antibacterial activity. The silver nanoparticles showed antibacterial activity on both gram-negative bacterium *Escherichia coli* (5.5 ± 0.2 mm to 6.5 ± 0.3 mm) and gram-positive bacterium *Micrococcus luteus* (7 ± 0.4 mm to 7.7 ± 0.5 mm). Our study is meaningful as a first observation indicating the possibility of using special plant organs to control the characteristics of nanoparticles.

## Introduction

Nanotechnology is a science that deals with the manipulation and fabrication of nanoparticles^1^. At least one or two dimensions of nanoparticles are within the range of 100 nm or less^2^. The nanometer-scaled particles have a unique property that differs them from their counterpart bulk material^3^, their small size offers a large surface-to-volume ratio which causes a substantial biochemical and catalytic activity compared to the particles with the same composition^1,4^. Nanoparticles are employed in the areas of drug delivery, biomedical sciences, gene delivery, chemical industries, optics, mechanics, catalysis and etc.^5^. Among metal nanoparticles, silver nanoparticles (AgNP) garner much attention due to their strong antibacterial and anti-inflammation effect. AgNPs are utilized in various physical, biological and pharmaceutical fields, for instance, cream or ointment containing AgNPs are applied for burn and wounds to inhibit bacterial infection^6^. Although AgNP is integrated into many areas, the exact mechanism explaining the particle formation is not fully uncovered yet. The traditional method for the synthesis of AgNP is to use physical and chemical approach to produce nanoparticles with controlled and well-defined size and shapes^7^. However, the use of toxic substances, high pressure and energy such as laser ablation, hydrothermal synthesis, solvothermal synthesis, pyrolysis and inert gas condensation brought a demand for more biologically compatiblenanoparticles^8,9^. Recently, the synthesis of AgNP through biological method has been studied intensely. The biological method offers nanoparticles with high yield and stability compared to the conventional physical and chemical approach^10^. AgNP can be biosynthesized by bacteria, fungi, yeast, actinomycetes and plant, thus avoiding the use of toxic substances and enabling for further application in medical and pharmaceutical field^11^. The application of plants for the synthesis of AgNP has gained significant attention. Plant-mediated synthesis of AgNP has many advantages, it can be obtained under ambient temperature with low cost and the process is relatively fast compared to bacteria, where a long process of maintaining cell culture is required^12^. Plants contain a wide range of metabolites that can aid in reducing silver ion, stabilizing and capping AgNP^13^, therefore the concentration and composition of AgNP will vary depending on the plant type^3^. This is especially the case for the medicinal plant as it is a rich source of complex phytochemicals and antioxidants. The main antioxidants of medicinal plants are polyphenols, carotenoids, and vitamins. The medicinal plants display a wide range of anti-inflammatory, antibacterial, antiviral, anti-aging, and anti-cancer activities^14^. In addition to polyphenols found in plants, there are other biomolecules responsible for reducing and capping AgNP^15^, these include polysaccharides, aldehydes, ketones, proteins, enzymes, amino acids, and caffeine^16^. The complex biomolecules found in medicinal plant assist in the reduction of metal ions and stabilization of nanoparticles into desired shape and size^17^. The plant-mediated synthesis of AgNP is relatively simple as it requires only plant extract and silver salt, thereafter it undergoes a reduction process^18^. There are many reports published regarding a medicinal plant-mediated synthesis of AgNP, these include *Gmelina aroberea* ^19^, *Tecomella undulata*^20^, *Artemisia absinthium*^21^, *Datura stramonium*^22^, *Calliandra haematocephala*^23^, *Carica papaya*^24^ etc. *Carduus crispus* is a plant species of the family Asteraceae that can be found in Mongolia. The medicinal effect ranges from a stomachache, rheumatism, atherosclerosis to cancer. And due to its medical properties, it is broadly applied in Mongolian traditional medicine^25^. The main activity of *Carduus crispus* is coagulation, antioxidant and anticonvulsive activity^26^. According to the study done by Baumberger the major compounds detected in *Carduus crispus* are flavonoids and coumarins, also alkaloids saccharides, essential oil, rubber, lipids contained in small quantities^27^. There are no available reports on the synthesis of AgNP using *Carduus crispus* as the plant extract. The mechanism of action of AgNP is not yet completely understood, however there are several hypotheses available explaining the antibacterial, anti-inflammatory and anti-cancer activity. It is known that nanoparticles have a large surface area that either penetrates the cell or attaches itself to the cell wall^11^, causing a disturbance in the membrane permeability making it porous^28^, and this action leads to a further leakage of cell content. Moreover, the appearance of pores on membrane result to diffusion of nanoparticles into the cell where it binds with sulfur and phosphorus-containing proteins, thus leading to the inactivation of proteins and DNA^17^. Another hypothesis suggests that the antibacterial activity of AgNPs results from the release of Ag^+^ ions through the oxidation dissolution process. Silver ions oxidized from AgNP mainly interact with thiol groups of various enzymes and protein, thereby interfering with the respiratory chain and disrupting the bacterial cell wall. Silver ions also facilitate the generation of reactive oxygen species (ROS), which is considered as the main cause for most cell death through the inactivation of DNA replication and ATP production^29^. The present study aimed to synthesize silver nanoparticles with medicinal plant *Carduus crispus* extracts and characterize the final product, and evaluate their antibacterial activities.

## Results and discussion

### UV–Vis spectra analysis and color change

**Figure 1:**
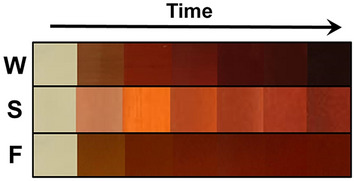
Color changes in biosynthesized silver nanoparticle with different parts of *Carduus crispus*. S-stem, F-flower and W-whole plant.

**Figure 2:**
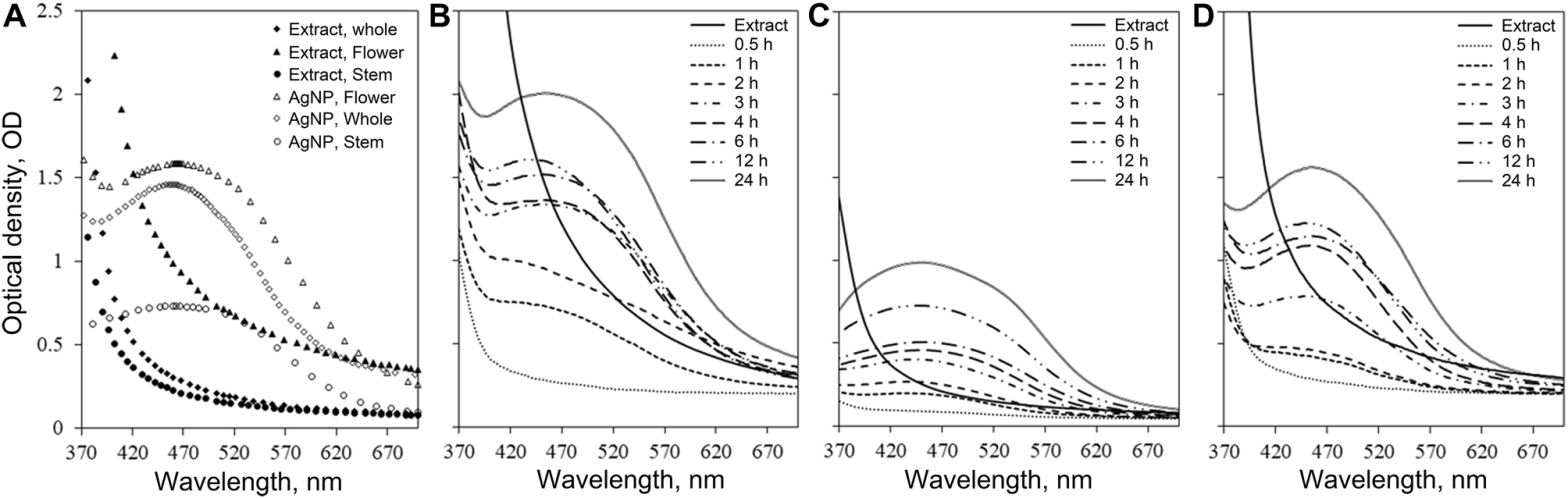
UV–Vis spectra for the reaction mixture containing of silver nanoparticles synthesized from *Carduus crispus* flower (AgNP-F), stem (AgNP-S) and whole plant (AgNP-W). Shown are the UV–Vis absorption spectra from 370 to 700 nm of all plant organs and synthesized (**A**) AgNPs, (**B**) AgNP-W, (**C**) AgNP-S, and (**D**) AgNP-F.

The visual color change from pale yellow to dark brown in response to time can be seen as evidence of silver ion reduction to AgNP. The change in color of biosynthesized AgNP is due to the excitation of surface plasmon resonance (SPR). Several studies done on the synthesis of AgNP via medicinal plant suggest the absorption peak around 412–470 nm with the duration of synthesis from 4 h till 24 h, these include medicinal plants, such as *Abutilon indicum, Aegle marmelos, Azadirachta indica, Calliandra haematocephala, Calotropis procera, Carica papaya, Helicteres isora, Lawsonia inermi, Leptadenia reticulate, Rheum palmatum, Tecomella undulata, Tagetes erecta, Urtica dioica*. The rate of color change from light yellow to dark brown varied in these studies, the earliest color change began within 1 h till 4 h^4,20,23,24,30–38^. Alternatively, different studies utilizing non-medicinal plants for the AgNP synthesis, such as *Allium cepa, Chenopodiastrum murale, Cyperus rotundus, Eleusin indica, Euphorbia hirta, Melastoma malabathricum, Musa acuminate, Pachyrhizus erosus, Rubus glaucus* exhibited absorption peak from 401–780 nm and was synthesized for 72 h till 14 days. The color change of AgNP synthesized via *C. murale* turned to brown color after incubating overnight^39–43^. The difference in color change rate might be due to the different properties of the plant, specifically, the medicinal plant contains a wide range of phytochemicals, such as flavonoids, polyphenols, terpenoids, etc.^44^ that assist in the formation of silver nanoparticles. Iravani et al.^5^ reported in their studies that flavonoids, polyphenols, terpenoids, alkaloids and proteins are the main constituents responsible for the reduction and stabilization of silver nanoparticles. Figure 1 shows the result of color change of the synthesized silver nanoparticle with different organs of *Carduus crispus*, such as stem, flower and the whole plant. It can be seen that different plant organs affected differently on silver nanoparticle synthesis, and particularly whole plant extract facilitated better silver nanoparticle formation compared to the stem and flower extract. The synthesis of silver nanoparticles with whole plant extract exhibited a darker color change. The variation in color change might be due to the different phytochemical content in the plant organs. Following the visual color change study, the formation and stability of silver nanoparticles synthesized with flower, stem, and whole plant of *Carduus crispus* were characterized using a UV–Vis spectrophotometer (Fig. 2). The results revealed that silver nanoparticles synthesized with whole plant (AgNP-W) exhibited higher absorption compared to silver nanoparticles synthesized using plant organs such as flower (AgNP-F) and stem (AgNP-S). The higher absorption is directly proportional to the higher yield of silver nanoparticles in colloidal solution^45^. Additionally, the size of the synthesized silver nanoparticle was studied by observing the shift of the absorption peak towards a longer or shorter wavelength^8,46^. In Fig. 2 b-d, silver nanoparticles were measured at various times, and according to our results, the AgNP-W exhibited blueshift in contrast to AgNP-F and AgNP-S, which can be interpreted as the formation of smaller-sized silver nanoparticles.

### Zeta potential analysis

**Figure 3:**
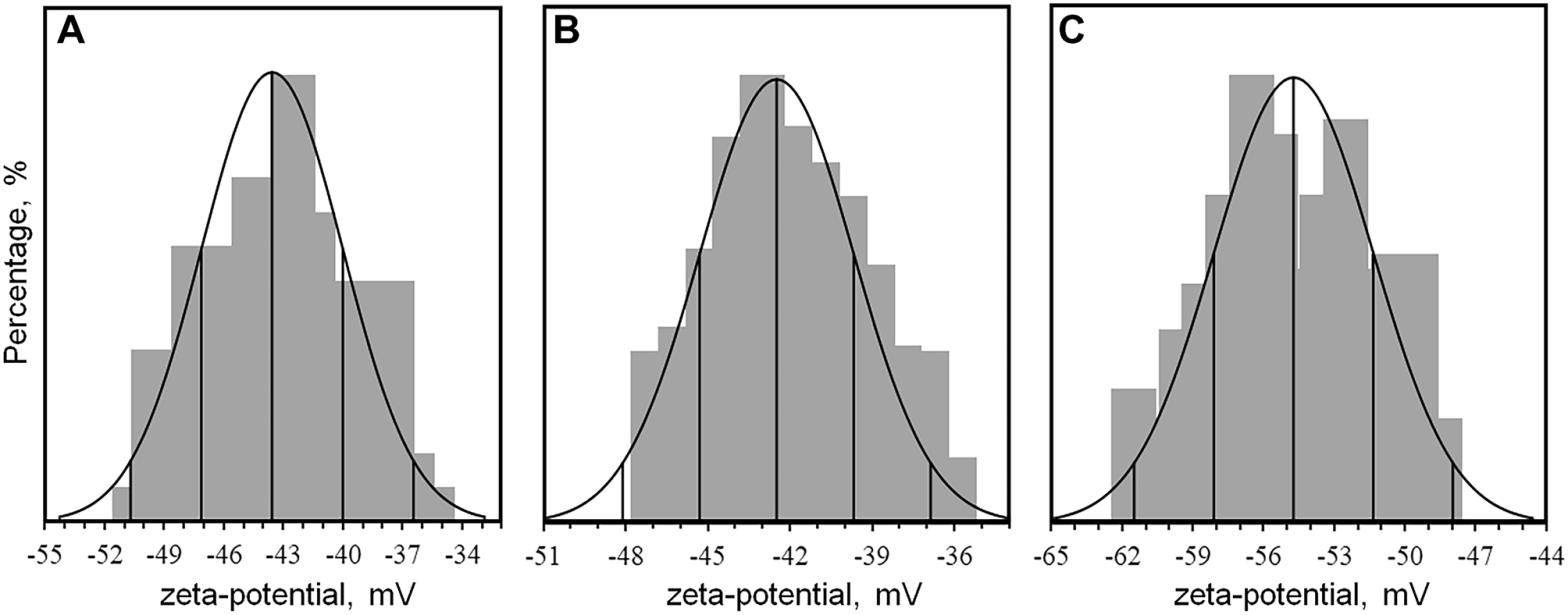
Zeta potential analysis of (**A**) AgNP-W, (**B**) AgNP-F and (**C**) AgNP-S.

Zeta potential explains the stability, dispersion and surface charge of the nanoparticles. The zeta potential greater than + 30 mV or less than − 30 mV indicates high stability of nanoparticles in dry powder form^31^. The high negative value produces repulsion between similarly charged particles in suspension, therefore resisting aggregation^47^. Several studies were done on silver nanoparticle synthesis with a medicinal plant such as Pot*entilla fulgens, Alpinia calcarata, Pestalotiopsis micospora, Urtica dioica, Jatropha curcas* which resulted inzeta potential of − 18 mV, − 19.4 mV, − 35.7 mV, − 24.1 mV, and − 23.4 mV respectively^4,6,12,47,48^. Our results showed that zeta potential of the synthesized AgNP-W, AgNP-S, AgNP-F had an average zeta potential of − 46.0 2 ± 4.17 (AgNP-W), − 54.29 ± 4.96 (AgNP-S) and − 42.64 ± 3.762 (AgNP-F) (Table 1). The zeta potential of AgNP-S exhibited a higher average value compared to the AgNP-W and AgNP-F, this may be due to the presence of different phytochemicals in each sample that reduces and cap silver nanoparticles. The results of the zeta potential analysis suggest that silver nanoparticles synthesized with *Carduus crispus* exhibit high stability and resist agglomeration. Figure 3 showed that zeta potential values of AgNP-W, AgNP-S, and AgNP-F fall within the normal distribution curve, which indicates that synthesized silver nanoparticles are fairly monodisperse.

**Table 1:** Average zeta potential and mobility of AgNP-W, AgNP-S and AgNP-F.

	Average zeta potential	Average mobility
AgNP-W	− 46.0 2 ± 4.17	− 3.52 ± 0.31
AgNP-S	− 54.29 ± 4.96	− 4.19 ± 0.38
AgNP-F	− 42.64 ± 3.762	− 3.25 ± 0.28

### FTIR spectral analysis of synthesized AgNP by Carduus crispus

**Figure 4:**
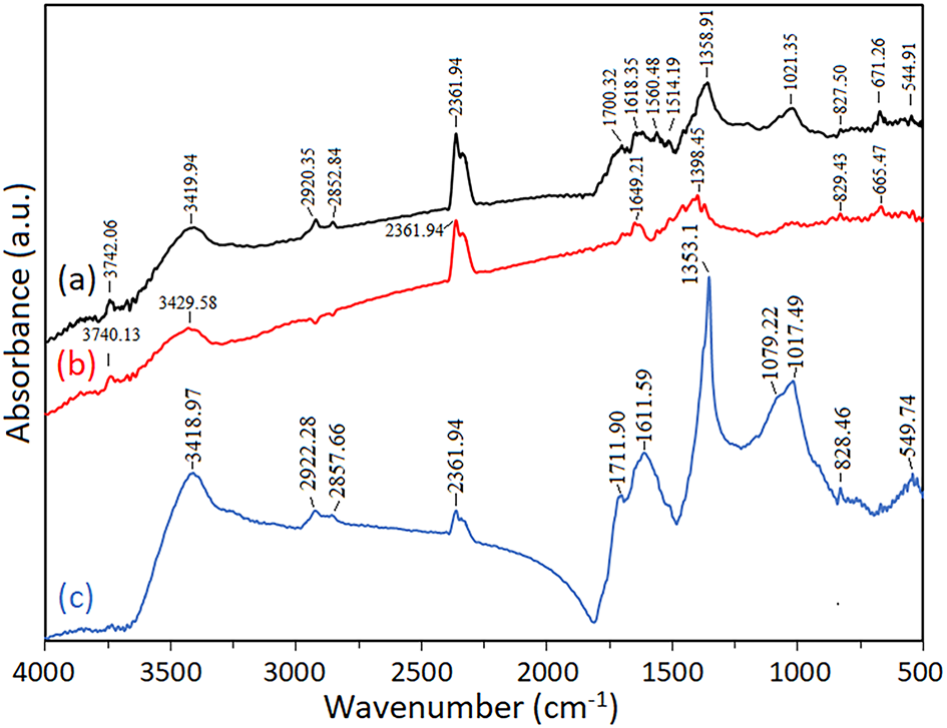
Fourier transform infrared spectra of (**a**) AgNP-W, (**b**) AgNP-S, and (c) AgNP-F.

The presence of the functional groups capping AgNP synthesized using *Carduus crispus* is analyzed by FTIR and shown in Fig. 4. The presence of various organic compounds in the plant reveals multiple peaks compared to the chemical method where only a few and strong peaks are displayed^49,50^. The results of our FTIR analysis showed the presence of several functional groups in AgNP-W, AgNP-S, AgNP-F. Additionally, the functional groups in AgNP-F and AgNP-S were present in AgNP-W samples as well, this may be attributed to the various phytochemicals capping the silver nanoparticles that are found both in flower and stem of *Carduus crispus*. The strong characteristic bands at ~ 3418 cm^−1^ to 3429 cm^−1^ and 2361 cm^−1^ in all samples AgNP-S, AgNP-F, AgNP-W are assigned to the O–H stretching/N–H stretching of amides and 2361 cm^−1^ to the C≡C stretching. Additionally, the weak band at ~ 1017 cm^−1^ to 1022 cm^−1^ and ~ 828 cm^−1^ assigned to carbohydrates and –C = O bending were found in all samples AgNP-S, AgNP-F, and AgNP-W. C–O stretching is present in AgNP-F which was observed from the very strong band at 1353 cm^−1^. The weak bands at 2922 cm^−1^ and 2857 cm^−1^ of CH_3_ stretch of alkane/carboxylic acids present in AgNP-F and were absent in AgNP-S. The band detected at ~ 3418 cm^−1^ to 3429 cm^−1^ and 1618.35 cm^−1^ correspond to the presence of phenolic compounds and flavonoids, and the band found on 1021.35 cm^−1^ indicates carboxylic acid, ester, and ether groups of proteins and metabolites that may be involved in the synthesis of nanoparticles^33^. Our result show that the strong band detected at 1611 cm^−1^ and 1017 cm^−1^ from AgNP-F correspond to the presence of flavonoids and proteins. On the other hand, weak bands detected at ~ 1696 cm^−1^ to 1371 cm^−1^ correspond to alcohol, carboxylic acids, alkyl halides/carboxylic acids/ester, alkenes/alkyl halides/aromatics, alkynes/alkyl halides stretch that peaks found from AgNP-S. According to Baumberger^27^ the major compounds detected in *Carduus crispus* are flavonoids and coumarins, in addition, alkaloids, saccharides, essential oil, rubber and lipids contained in small quantities which is in line with the presence of flavonoids and phenolic compounds in our synthesized AgNP. The AgNP-F and AgNP-S contained different functional groups that correspond to various compounds, and AgNP-F revealed that it has a strong correlation with flavonoids from *Carduus crispus*. The results of FTIR and UV–Vis spectra analysis confirm that these functional groups are the capping and reducing agents responsible for the synthesis of AgNPs.

### XRD, PCCS, SEM/EDX and AFM analysis

**Figure 5:**
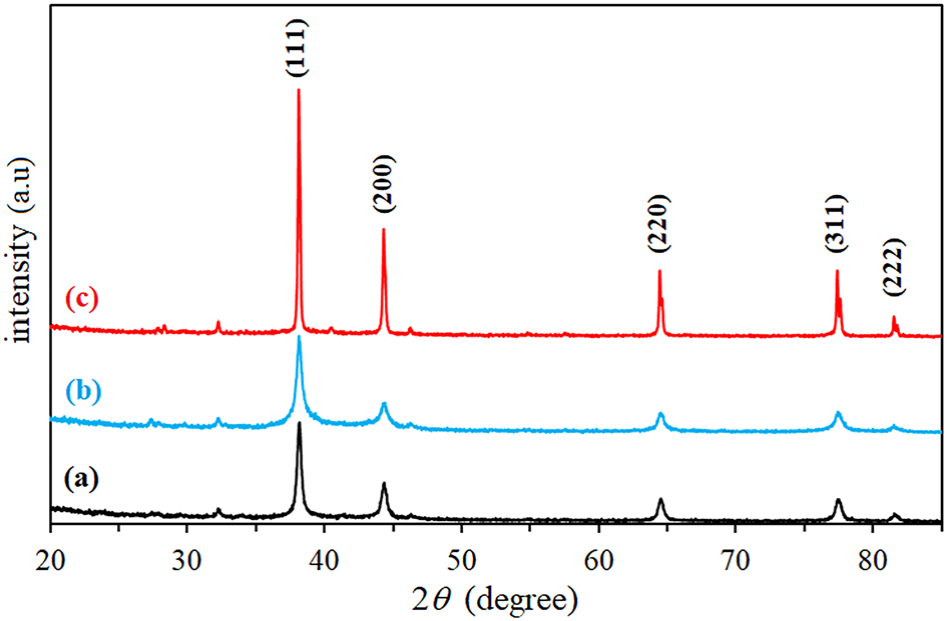
XRD spectra of (**a**) AgNP-W, (**b**) AgNP-F, (**c**) AgNP-S. Peaks are appeared at 111, 200, 220, 311 and 222.

**Figure 6:**
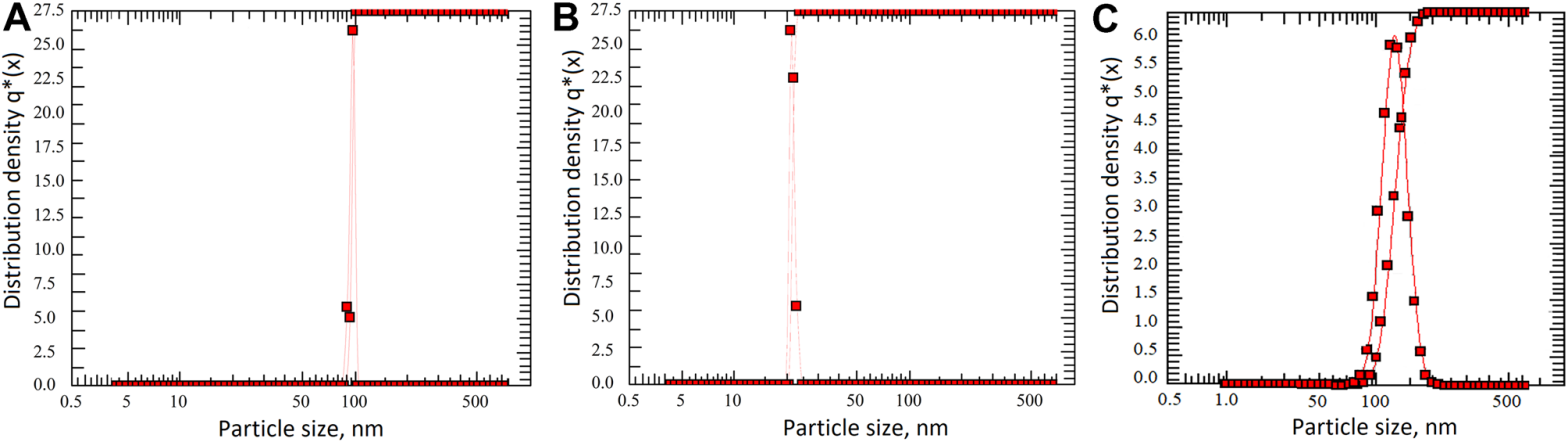
PCCS analysis: particle number distribution of synthesized AgNP-W (**A**), AgNP-F (**B**) and AgNP-S (**C**).

**Figure 7:**
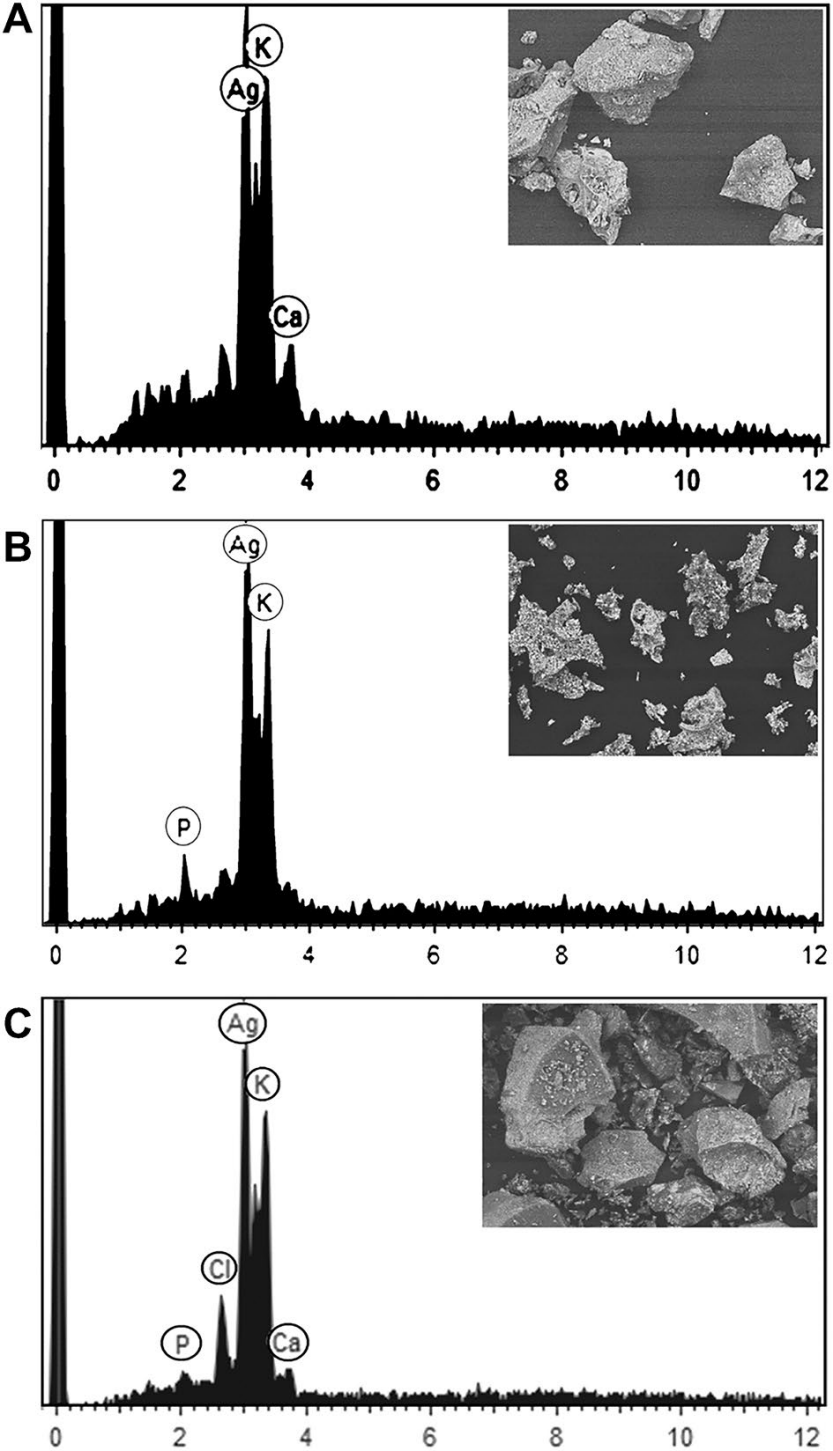
EDX spectra for (**A**) AgNP-F, (**B**) AgNP-S and (**C**) AgNP-W along with SEM image area (inset).

**Figure 8:**
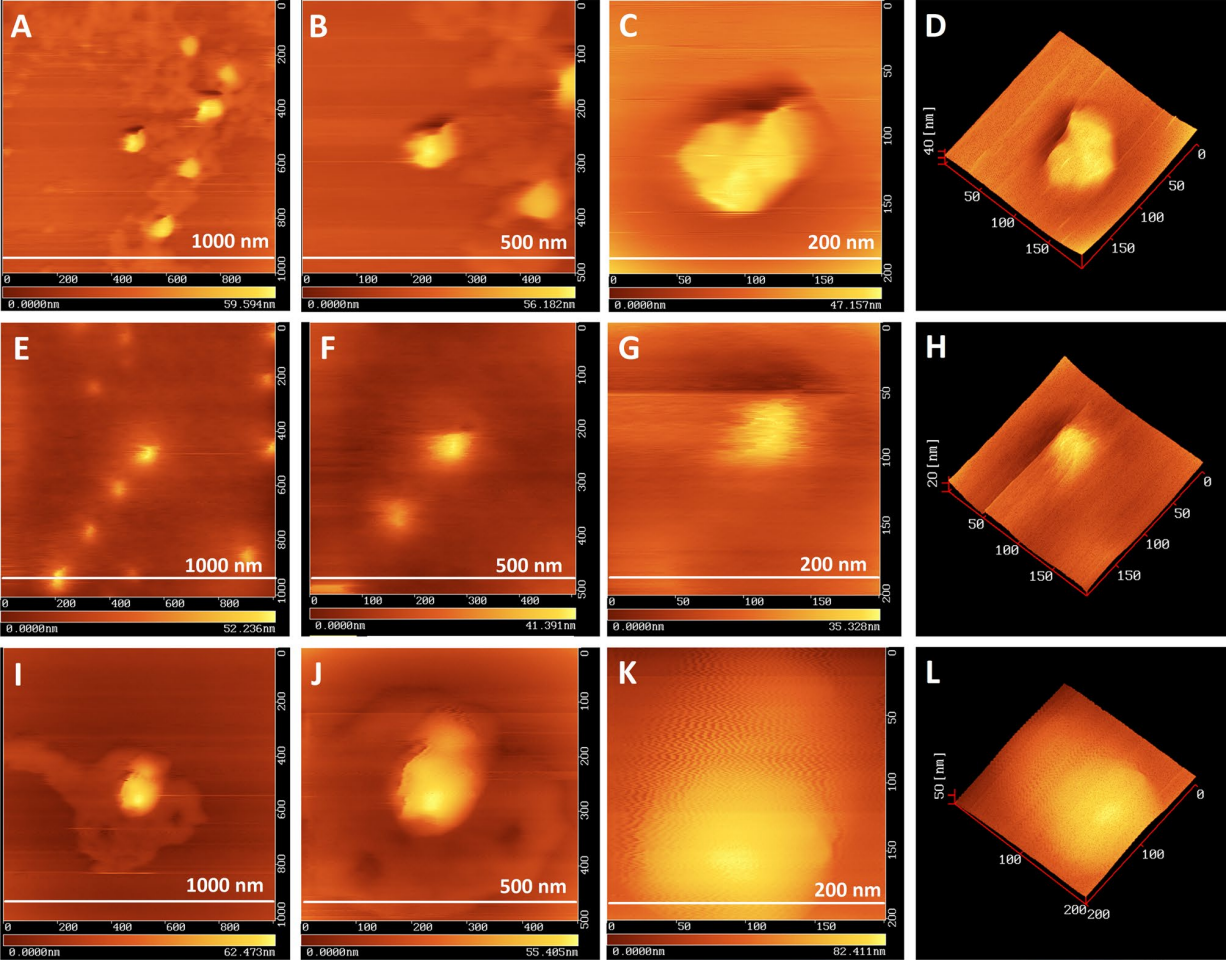
Atomic force microscopy images (2D and 3D) of silver nanoparticles on siliconized cover slide; AgNP-W (**A**–**D**), AgNP-F (**E**–**H**) and AgNP-S (**I**–**L**).

The crystalline nature of the synthesized AgNP was confirmed by X-ray crystallography. The XRD pattern of the nanoparticles was analyzed with an XRD instrument and shown in Fig. 5. Bragg reflection of the 2θ peaks was observed at 32.25˚ to 81.62˚ and corresponded to (111), (200), (220), (311), (222) plane lattice which can be indexed to the face-centered cubic crystal nature of the silver. The average crystallite size was calculated using the Scherrer equation. The average crystallite sizes were 13 nm (AgNP-F), 14 nm (AgNP-W) and 36 nm (AgNP-S). The results of our study are in line with other published literature, the crystal nature of silver nanoparticles synthesized with *Tagetes erecta* ^31^, *Urtica dioica*^4^, *Aegle marmelos* was face-centered cubic with diffraction peaks of (111), (200), (220), (311) respectively ^34^. PCCS is a technique based on the Brownian motion that measures the average nanoparticle size (grain size). In Fig. 6, the average particle size of AgNP-W, AgNP-F and AgNP-S was 99.6 nm, 22.5 nm and 145.1 nm respectively. The difference between PCCS and XRD analysis lies in the measurement method of the particle. Application of the Scherrer equation on XRD data gives the average crystallite size, specifically the size of a single crystal inside the particle or grain. The morphological and elemental analysis was done on Scanning Electron Microscope (SEM) and Energy Dispersive X-Ray Spectroscopy (EDX). The elemental composition of the synthesized silver nanoparticle was assessed using EDX spectroscopy (Table 2). The results in Fig. 7 showed that AgNP-W, AgNP-S, and AgNP-F contained silver and potassium elements together with several other elements that differed in AgNP-F and AgNP-S samples, i.e. AgNP-F included phosphorus 2.8%, potassium 15.2%, and AgNP-S had calcium 7.5%, pottassium 15.5% elements. In contrast, AgNP-W contained all the elements including the elements that differed in AgNP-F and AgNP-S. Interestingly, the silver element in AgNP-F had the highest content of 82% compared to AgNP-W and AgNP-S which had a silver content of 79% and 77% respectively. Another observation on EDX analysis revealed that AgNP-W, AgNP-F, AgNP-S did not show the presence of nitrogen peak, this indicates that trace ions from AgNO_3_ are absent in the samples. The size of biosynthesized AgNP-W, AgNP-F and AgNP-S was determined with Atomic Force Microscopy (AFM). Figure 8 show that the size of nanoparticles differed, for instance, AgNP-W had a size of 70 nm, AgNP-F with size 33 nm and AgNP-S with size 131 nm. Figure 8 (A-C, E–G and I-K) represents the two dimensional images of AgNP-W, AgNP-F and AgNP-S. Figure 8 (D, H and L) shows the three dimensional image of AgNP-W, AgNP-F and AgNP-S respectively. The different composition of plant organs, such as stem, flower and whole plant could be the reason for the observed variability in, color change, UV–Vis absorption, EDX, FTIR. In addition, the results of AFM data and XRD show that the synthesis of AgNP can be manipulated with different plant organs.

**Table 2:** Elemental composition of the synthesized silver nanoparticles by *Carduus cripus.*

	Silver, %	Potassium, %	Calcium, %	Phosphorus, %	Chlorine, %
AgNP-W	77 ± 1	15.1 ± 0.5	2.8 ± 0.1	1.2 ± 0.1	3.9 ± 0.21
AgNP-F	82 ± 1	15.2 ± 0.4	–	2.8 ± 0.15	–
AgNP-S	77 ± 1	15.5 ± 0.5	7.5 ± 0.2	–	–

### Antibacterial activity

**Figure 9:**
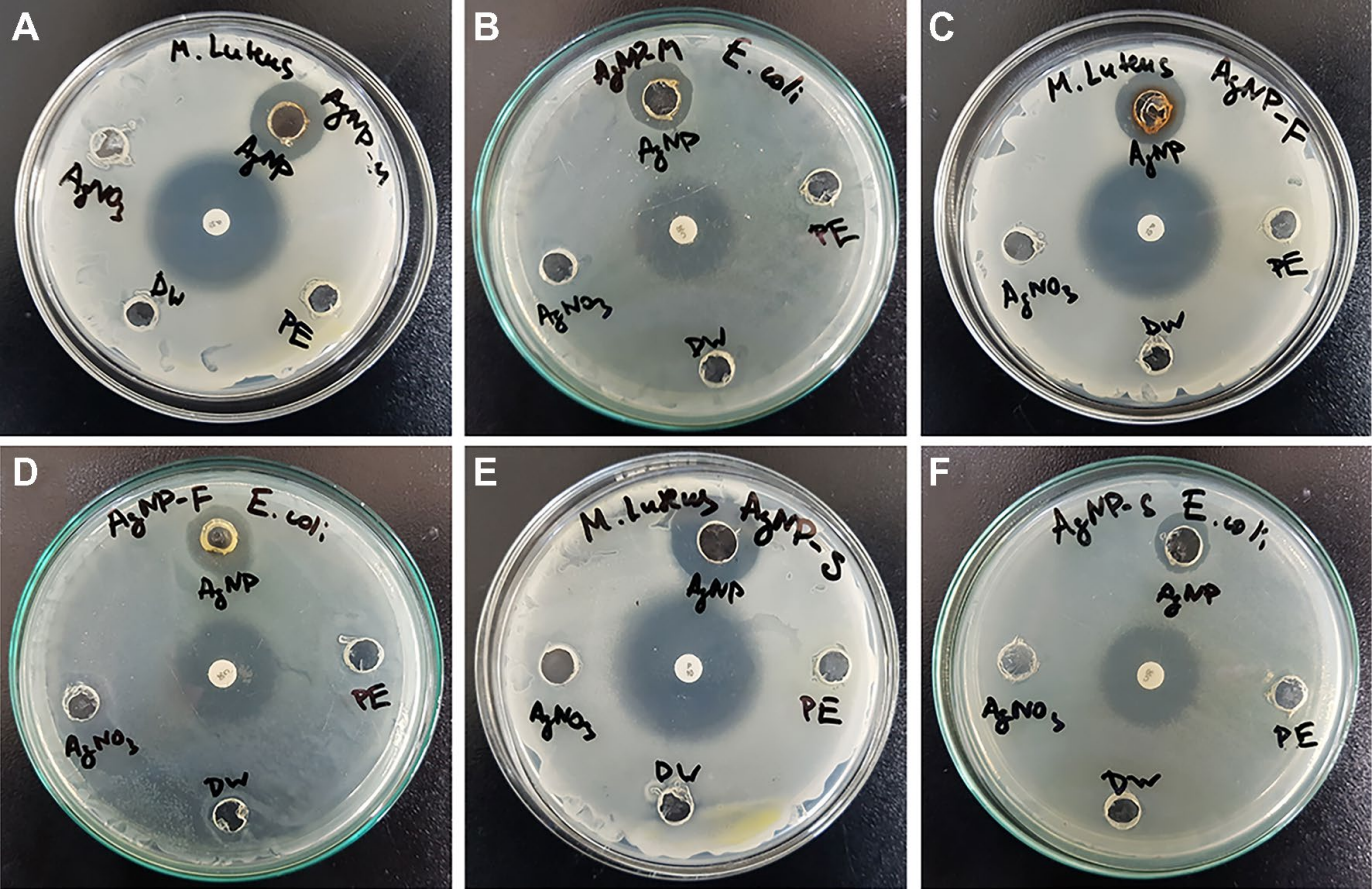
Petri dishes showing the zone of inhibition of synthesized AgNP-W on (**A**) *M. luteus* and (**B**) *E. coli*, and AgNP-F on (**C**) *M. luteus* and (**D**) *E. coli*, AgNP-S on (**E**) *M. luteus* and (**F**) *E. coli* (AgNP: silver nanoparticle, AgNO_3_: silver nitrate, DW: distilled water, PE: plant extract).

The antibacterial activity of silver nanoparticles was studied against pathogenic bacterial strains of gram-negative *E.coli* and gram-positive *M.luteus* using the well diffusion method (Fig. 9). Standard antibiotics such as Penicillin G and Chloramphenicol, plant extracts, AgNO_3_ and distilled water were chosen as the control group. The results of the antibacterial activity showed that all synthesized silver nanoparticles had efficient antibacterial activity against both gram-negative *E.coli* and gram-positive *M.luteus* bacterial strains. The inhibition zone of AgNP-F, AgNP-W and AgNP-S against *E.coli* and *M.luteus* were 6.5 ± 0.3, 6 ± 0.2, 5.5 ± 0.2 and 7.5 ± 0.3, 7 ± 0.2, 7.7 ± 0.4 mm respectively. The plant extract and AgNO_3_ did not reveal any antibacterial activity against both *E.coli* and *M.luteus*, which can be interpreted that AgNP-W, AgNP-F, and AgNP-S are solely responsible for the antibacterial activity. The mode of action of AgNPs against bacteria is not completely understood yet. However, several hypotheses are explaining the antibacterial activity of silver nanoparticle: (1) generation of reactive oxygen species; (2) release of Ag + ions from AgNPs denaturize proteins by bonding with sulfhydryl groups; (3) attachment of AgNPs on bacteria and subsequent damage to bacteria^4,11,24^. The multiple published reports on the antibacterial activity of silver nanoparticles against gram-negative and gram-positive bacteria showed that silver nanoparticles had a slight antibacterial activity on gram-positive bacteria^6,22,31,36^. Interestingly, AgNP synthesized by *Carduus crispus* exhibited effective inhibition on both gram-positive and gram-negative bacteria which can be interpreted that the antibacterial activity of silver nanoparticles (AgNP-W, AgNP-F and AgNP-S) is not affected by the difference in the bacterial wall.

### In-vitro cytotoxicity assay

**Figure 10:**
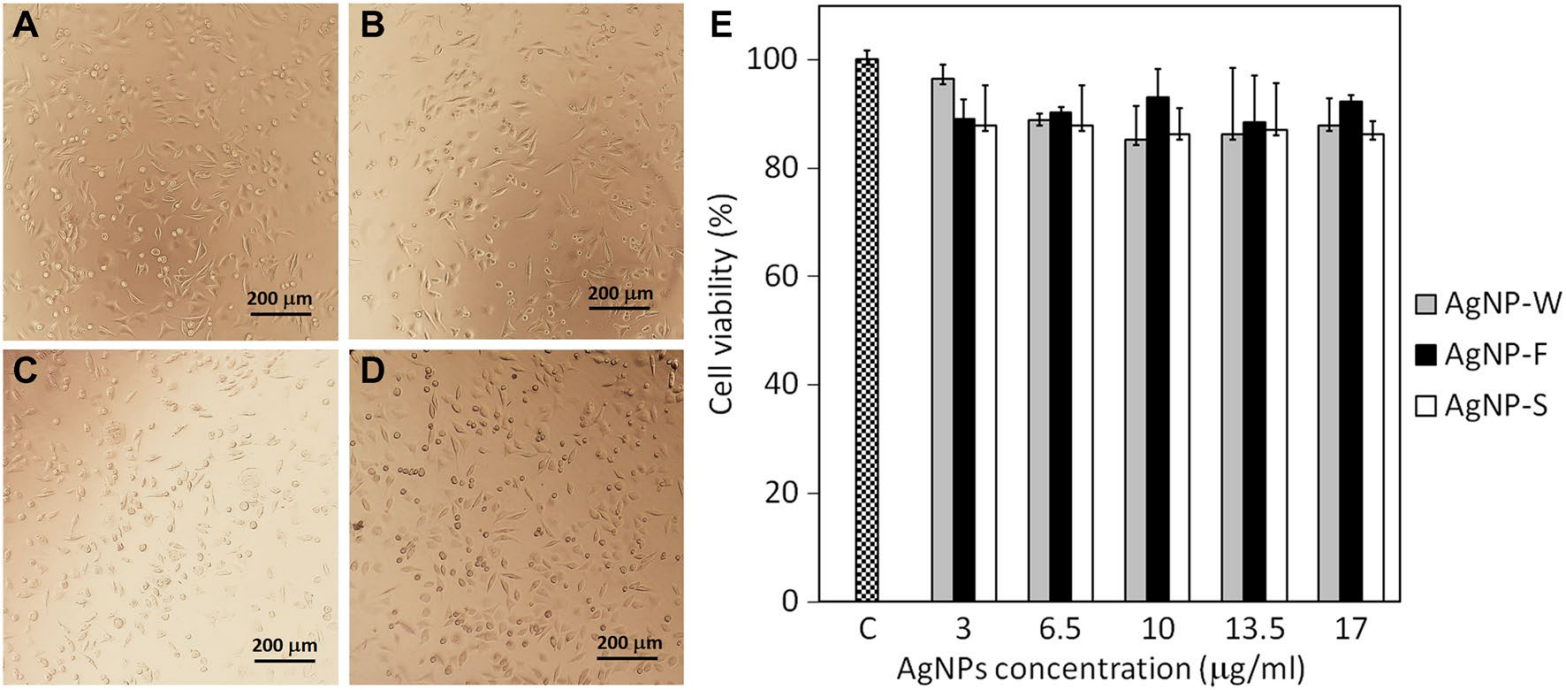
A microscopic pictures of HepG2 cells treated with AgNPs for 24 h in cell culture: control (**A**), AgNP-W (**B**), AgNP-F (**C**) and AgNP-S (**D**). After 24 h, the cell toxicity effect was examined with Crystal Violet (**E**).

Cytotoxicity is considered as an important indicator for cell viability, therefore in this study we employed crystal violet assay to investigate the effect of different concentration of AgNP-W, AgNP-F and AgNP-S on the adherent human hepatoma cell line HepG2 (Fig. 10). The liver is an important organ with detoxifying effect, additionally, it is considered as an accumulation site for AgNPs^51^. In this study, the untreated HepG2 cell lines revealed significant adherence to the well plate. On the other hand, the treated cells with nanoparticles exhibited small decrease in cell viability after 24 h incubation at 3 to 17 µg/ml. The cell viability of these treated groups with AgNP-W, AgNP-F and AgNP-S were 87.93 ± 4.87%, 92.24 ± 1.21% and 86.20 ± 2.43% at 17 µg/ml. The toxicity of AgNPs to bacteria and human cells is widely known, however, the result of our study suggests that AgNPs synthesized by medicinal plant *Carduus crispus* with concentration of 3 to 17 µg/ml have low toxicity on HepG2 cell line (Fig. 10A,B). In addition, biosynthesized silver nanoparticles possessed efficient antibacterial activity against Gram-negative and Gram-positive bacteria (Fig. 9). The antibacterial activity of the synthesized AgNPs and their low toxicity to human cells may enable further application in biomedical field. The low toxicity of biosynthesized AgNPs to adherent human cells are similar to other published reports^52^.

## Conclusion

The synthesis of silver nanoparticles via biological method, specifically plant extracts provides a natural, eco-friendly, cost-effective, rapid synthesis of silver nanoparticles. The present study reports the synthesis of silver nanoparticles with medicinal plant *Carduus crispus* in reducing silver ions and stabilizing the silver nanoparticles. It has been reported that medicinal plants are a rich source of phenolic compounds such as flavonoids and phenolic acids, etc. Additionally, plant organs contain different contents of phenolic compounds, therefore flower, stem, and whole plant of *Carduus crispus* were chosen for this study. Afterwards, the synthesized silver nanoparticles were characterized using visual color change, UV–Vis spectroscopy, zeta potential, FTIR, XRD, PCCS, SEM–EDX and AFM. The characterization of AgNP-W, AgNP-F, and AgNP-S revealed that AgNP-W had a higher yield, synthesis rate, and smaller-sized silver nanoparticles. The zeta potential conveys the stability and the result of all the synthesized silver nanoparticles showed the zeta potential value of − 46.0 2 ± 4.17 (AgNP-W), − 54.29 ± 4.96 (AgNP-S), and − 42.64 ± 3.762 (AgNP-F) which indicates highly stable silver nanoparticles. The variation in zeta potential may be due to the different phytochemical properties of the plant. Then FTIR analysis was utilized to study the role of phytochemical properties in plants for the synthesis of silver nanoparticles, the results showed that different functional groups in AgNP-F and AgNP-S were also present in AgNP-W samples as well. And based on the UV–Vis spectra analysis, AgNP-W and AgNP-F had the highest absorbance compared to AgNP-S, therefore we can conclude that the functional groups present and coincided in both AgNP-F and AgNP-W may play a contributing role in capping and synthesis of silver nanoparticles, these include functional groups with bands at 2922.28 cm^−1^, 2857.66 cm^−1^, 1711.90 cm^−1^, 1611.59 cm^−1^, 1079.22 cm^−1^ and 1017.49 cm^−1^ which correspond to alkanes, carboxylic acids, ketones, alkenes, amides, esters/ethers/amides, alkyl halides. Furthermore, strong bands at 3418 cm^−1^ to 3429 cm^−1^, 1618.35 cm^−1^ to 1611 cm^−1^, and 1017 cm^−1^ correlates to flavonoids and phenolic compounds. The EDX analysis detected the following elements, such as silver, potassium, phosphorus in AgNP-F; silver, potassium, calcium, chloride, and phosphorus in AgNP-W; finally, silver, potassium, calcium in AgNP-S samples. The synthesized silver nanoparticles had an average crystallite sizes of 14 nm (AgNP-W), 13 nm (AgNP-F) and 36 nm (AgNP-S) with face-centered crystal structure and average grain sizes of 99.6 nm (AgNP-W), 22.5 nm (AgNP-F) and 145.1 nm (AgNP-S). The sizes detected in AFM was 70 nm (AgNP-W), 33 nm (AgNP-F) and 131 nm (AgNP-S). Although the method of synthesis varied in AgNP-F, AgNP-W, and AgNP-S, their antibacterial activity showed efficient inhibition on both gram-negative and gram-positive bacteria. Based on these results, we can conclude that silver nanoparticles synthesized by whole plant of *Carduus crispus* have a faster rate of synthesis, higher yield with a smaller size, and high antibacterial activity against both gram-negative and gram-positive bacteria. The overall results show that the effectiveness of the synthesis of the flower for AgNP appears similar to using whole plant. Additionally, we have shown that the process of synthesizing nanoparticles can be manipulated with specific organs of plant, for example, particle size and synthesis duration, biological effect, etc. Our study is meaningful as a first observation indicating the possibility of using special plant organs to control the characteristics of nanoparticles. Moreover, further studies are required in this area.

## Methods

### Chemicals and plant

The *Carduus crispus* was collected from Khuder soums, Selenge province of Mongolia (GPS coordinates: N 49.641772, E 107.80935) and the taxonomy was determined by a botanist Kh.Khaliunaa from National University of Mongolia. The *Carduus crispus* used for the study does not violate the local regulations of Mongolia, the permission for the plant collection was granted from the Ministry of Environment and Tourism of Mongolia. The collected plant specimen of *Carduus crispus* was deposited into the publicly available herbarium of National University of Mongolia with deposition number UBU0002509. The Silver Nitrate (AgNO_3_) with ≥ 99.0% purity was purchased from Sigma Aldrich. All the other relevant reagents are up to the standard.

### Preparation of plant extract

The whole plant was washed with tap water in order to remove the adhering dust and soil particles, followed by washing with distilled water. 100 ml of distilled water was added to 5 g of *Carduus crispus* and boiled for 15 min, then cooled at ambient temperature. Afterward, it was filtered by Whatman filter paper and centrifuged twice at 10,000 rpm to obtain a plant extract. Finally, the extract was ready for the synthesis of AgNP.

### Synthesis of silver nanoparticle

The aqueous plant extract of *Carduus crispus* and AgNO_3_ (1 mM) were mixed with the ratio of 1:16, then the solution was exposed to the daylight and the reaction took place at the various time at room temperature. In order to obtain silver nanoparticles in powdered form, the solution was vaporized on a vacuum evaporator, and the final product of AgNP was kept inside the oven at a temperature of 300 °C for 4 h.

### Characterization of AgNP synthesized by Carduus crispus

AgNP was successfully synthesized by using *Carduus crispus*. A color change from pale yellow to colloidal dark brown indicated the formation of silver nanoparticles. UV–Vis spectra analysis offers an insight into the synthesis and stability of the AgNP. Formation of the biosynthesized AgNP was determined by the UV–Vis spectrophotometer (Shimadzu UV-2500PC Series) at 30 min, 1 h, 2 h, 3 h, 4 h, 6 h, 12 h, 24 h and was carried out at 350–700 nm range. FTIR spectrum was recorded in the range of 500 to 4000 cm^−1^ through the potassium bromide powder method using FTIR spectrophotometer (Prestege-21, Shimadzu, Japan) for understanding the constituent capping and reducing agents of silver nanoparticles. Also, elemental composition of the synthesized silver nanoparticles was analyzed with an energy dispersive X-ray spectroscope instrument (TM-10000 with EDX). To identify the structural phase present in the AgNP, XRD was performed by XRD instrument (Shimadzu, Maxima-X-7000) operating at 40 kV with a current of 30 mA and Co-Ka radiation. And crystalline size was determined by Scherrer equation. In order to understand the size distribution and surface charge, the zeta potential (ZetaCompact, CAD Instruments, France) and Photon Cross-correlation Spectroscopy (PCCS) (NANOPHOX 1 nm to 10,000 nm, Sympatec GmbH, Germany) methods was used for dispersed nanoparticles of silver.

### Atomic force microscope (AFM) measurement

The size of the synthesized AgNP was analyzed with Atomic Force Spectroscopy (SPA 300, Seiko Inc., Japan). First, the siliconized glass cover slides selected for the AFM measurement were immersed in ultra-pure water and sonicated for 10 min with ultra sonicator, afterwards the siliconized glass cover slides were rinsed with ethanol solution and air-dried in laminar box at RT, then the samples for AFM analysis were prepared by drying the AgNP suspension on prepared siliconized glass cover slide at RT. Finally, Atomic Force Microscope was used to analyze the morphology and size of the samples via golden silicon probe (GSG11) with tip curvature radius of 10 nm.

### Determination of anti-bacterial activity using well diffusion method

The agar well diffusion method was used to study the antibacterial activity of the synthesized silver nanoparticle. Broth medium was used to subculture bacteria and was incubated at 37 °C for 24 h, afterwards, overnight cultures were taken and spread on the agar plates to cultivate a uniform microbial growth plate. The bacterial strains were gram-negative *Escherichia coli* and gram-positive *Micrococcus luteus*. And silver nitrate, plant extract, antibiotics (Penicillin G against *Micrococcus luteus* and Chloramphenicol against *Escherichia coli*) were chosen as the control group for the study of antibacterial activity. Finally, the petri dishes were incubated for 24 h at 37 °C. In order to evaluate the antibacterial activity of the synthesized silver nanoparticle, the diameter of the inhibition zone was measured and compared with the control groups.

### Cell culture

The cell line HepG2 was cultured in Dulbecco’s Modified Eagle Medium (DMEM) medium supplemented with 10% Fetal Bovine Serum (FBS), 1% penicillin, 1% streptomycin and maintained in standard condition with 5% CO_2_ humidified incubator at 37 °C temperature. Prior treatment, the cells were seeded in 96-well tissue culture plates with a seeding density of 2 × 10^4^ cells and incubated overnight. The cell line HepG2 were sub-cultured at 70–80% confluence and used for further study.

### Crystal violet assay

The crystal violet assay was performed to determine the cell viability according to the method described by Feoktistova et al. Crystal violet is a dye which binds to DNA and protein of cells and used for studying viable cells that are adhered to the cell culture plates. In order to study the cytotoxicity of the synthesized AgNP-W, AgNP-F and AgNP-S, the nanoparticles were first filtered with 0.45 µm filter. Different concentration of AgNP-W, AgNP-F and AgNP-S suspension (3–17 μg/ml) were added to triplicate well and incubated for 24 h. After treatment the medium was removed and the cells were washed twice with PBS, followed by staining with 50 µl of 0.5% crystal violet dye for 20 min at room temperature. Thereafter, crystal violet dye was removed and the wells were washed in a stream of tap water and left to air-dry for 3 h at room temperature. The crystal violet dye was dissolved with the addition of methanol to each well and the absorbance was measured at 570 nm with ELISA reader.

### Statistical analysis

All experiments were performed at least three times, independently and data were analyzed by a Student’s t-test, and a value of p < 0.05 was considered significant.
